# Is non-operative treatment of acute appendicitis possible: A narrative review

**DOI:** 10.1016/j.afjem.2024.03.006

**Published:** 2024-04-08

**Authors:** Hani Bendib

**Affiliations:** a Department of General Surgery, EPH Kouba, Algiers, Algeria; b Faculty of Medicine, Algiers 1 University, Algeria

**Keywords:** Acute appendicitis, Appendectomy, Antibiotic therapy, Conservative, Review, Treatment

## Abstract

**Introduction:**

Acute appendicitis (AA) represents the most frequent surgical emergency. Perforation was long considered the ultimate outcome of AA, prompting appendectomy; which remains the standard treatment. New data have clarified the role of the appendix, justifying conservative treatment. This narrative review aims to summarize the evidence regarding the non-operative treatment (NOT) of AA in adults.

**Methods:**

The literature search was performed via the PubMed Medline database. Our criteria-based selection resulted in a total of 48 articles for review.

**Results:**

Recent trials and meta-analyses have assessed NOT, which support primary antibiotic treatment of uncomplicated AA. Although it has a significant recurrence and failure rate, NOT does not appear to increase the risk of appendicular perforation. Moreover, NOT compared with appendectomy, seems to be associated with less morbidity, lower cost of care and preserved quality of life.

**Conclusion:**

First-line NOT seems to be a reasonable approach for the treatment of uncomplicated CT-confirmed AA. Careful patient screening would definitely enhance the success rate.

**African Relevance**

The non-operative approach, could avoid many unnecessary appendectomies, along with their burden of non-negligible complications.

The non-operative treatment of uncomplicated appendicitis could be a useful option in low and middle income countries such as in Africa by preserving both human and material resources.

The conservative approach, with less morbidity and a shorter duration of physical disability, would significantly reduce healthcare costs and expenses in the African context.

Alt-text: Unlabelled box

## Introduction

Acute appendicitis (AA) is among the commonest causes of acute abdominal pain. In industrialized countries, AA incidence ranges from 5.7 to 50 cases per 100,000 inhabitants per year, reaching a peak between 10 and 30 years old [1]. In terms of lifetime risk, there are geographical variations. This risk is estimated to be 9 %, 8 % and 2 % respectively in the US, Europe and Africa [2]. Disease severity, the radiological assessment and treatment of AA are also conditioned by countries' resources [3]. The pathogenesis of AA is mainly based on the theory of luminal obstruction; leading to mucus accumulation and bacterial proliferation, which causes tension in the appendicular wall and may eventually lead to necrosis and perforation [4]. The diagnosis is based primarily on the patient's history, physical examination and inflammatory biomarkers. Right iliac fossa pain combined with tenderness or rebound are the best signs to conclude AA in adults [4]. Appendectomy, by open laparotomy and especially by laparoscopy, is still the standard of care for AA [5]. In the US, over 300,000 appendectomies are performed each year [4].

It is to the French surgeon, Claude Amyand that we owe the first report of an appendectomy in 1735. Operated for a hernia, the 11 year old boy had a perforated appendix located in the hernia sac [6]. In 1880, the Scotsman, Robert Lawson Tait reported the first appendectomy for suspected AA [7]. In 1883, it was the turn of the Canadian, Abraham Groves to perform the first appendectomy in North America [8]. A few years later, Charles Heber McBurney, an American surgeon published his first series, and described the surgical technique that still bears his name [9]. For over a century, appendectomy by McBurney incision remained the standard technique, until the first laparoscopic appendectomy was performed by Karl Semm in 1980 [10].

The non-operative approach to AA is probably not new. As early as 1910, Smith and Wood Jones reported the first case of presumed spontaneous resolution in an uneviscerated mummy of a young woman from Nubia, dating back to the Byzantine period [11]. A non-operative treatment (NOT) algorithm was first proposed by Bailey in 1930 [12]. Coldrey described in 1959, the first large series of NOT in 471 patients, with a failure rate of 10 % [13]. Both American and Soviet sailors serving on fishing vessels have also received offshore NOT in the 70′s and 80′s [14]. Chinese authors have reported a series of patients treated conservatively with traditional Chinese medicine and antibiotics for others; with a low failure rate [15]. However, these findings should be interpreted with some caution, due to the lack of both the diagnosis and the follow-up.

## Methods

***Search strategy:*** A broad literature search was conducted to assess the current evidence for the non-operative management of AA in adults. PubMed advanced search was completed in September 2022 using the term combination ((appendicitis[MeSH Terms]) AND (management[MeSH Terms])) AND ((antibiotics[MeSH Terms] OR (appendectomy[MeSH Terms])).

**Fig. 1:**
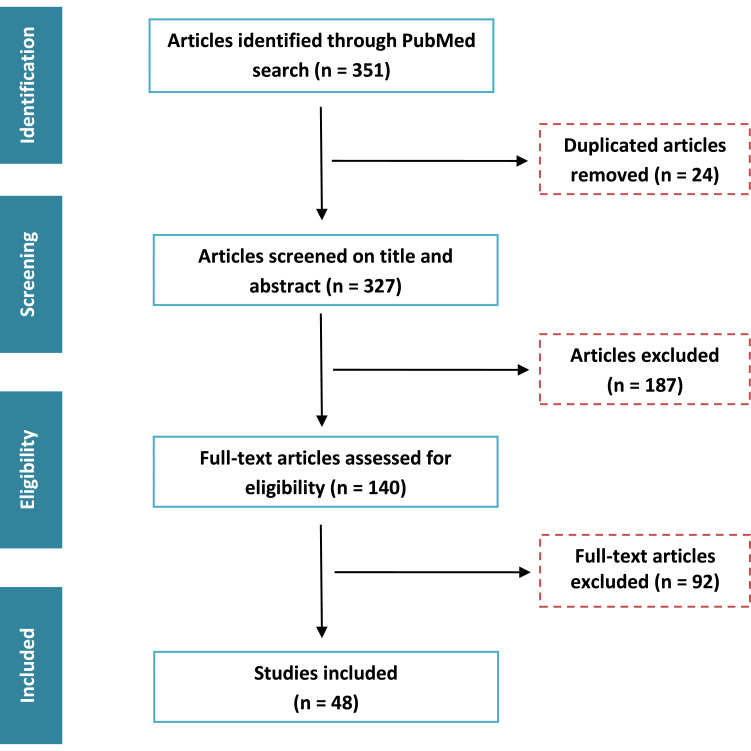
Flowchart describing the article selection process.

***Eligibility criteria and data collection:*** All studies dealing with the NOT in adults were included. Exclusion criteria were studies involving patients under 16 years old. No language or time restrictions were applied. Results from all searches were combined and duplicate data were removed. Articles were assessed according to our inclusion and exclusion criteria, and eligibility was evaluated by three reviewers (HB, ND and HO). A total of 48 papers were involved in this review. In addition, a deeper analysis of the cited references by the extracted articles was also included. Most resources included were guidelines, randomized controlled trials or meta-analysis. The flowchart for study selection is shown in Fig. 1.

## Discussion

### *Rational for conservative treatment*

Several reasons have been put forward to justify the non-operative approach. Indeed, the vermicular appendix in humans was long thought to have no major function. However, a number of recent studies suggest the opposite. According to De Coppi et al. [16] the appendix is able to produce mesenchymal stem cells. Under stimulus, these cells may be able to differentiate into other highly specialized cell types; suggesting the role of a stem cell reservoir used to repair or recolonize the intestine after a bacterial infection [17]. Furthermore, there appears to be a correlation between uncomplicated appendicitis incidence and the rate of negative appendectomies. This may finally suggest that many AA may have spontaneous resolution. In 1989, Luckman suggested that perforated and non-perforated appendicitis could be two distinct entities [18].

In addition, and despite the progress achieved in both surgery and intensive care, open or even laparoscopic appendectomy is still associated with a significant morbi-mortality [18]. In an American cohort of 261,134 appendectomies, Flum and Koepsell reported a 15.3 % rate of negative appendicitis, clearly more frequent in women [19]. Compared with AA, negative appendicitis was associated with increased morbidity (2.6% vs 1.8 %, *P* < 0.001), influencing the length of hospital stay, which was significantly longer (5.8 vs 3.6 days, *P* < 0.001); the lethality rate was also significantly higher (1.5% vs 0.2 %, *P* < 0.001). The same authors estimated at $741.5 million the total cost of managing these negative appendicitis [19]. These results corroborated those of the Swedish cohort of Blomqvist et al. [20] who reported a 30-day postoperative mortality three times higher for non-perforated appendicitis.

Several studies have shown an increase in the rate of intestinal occlusions after appendectomies up to 10 % [21], [22], [23]. More recently, Leung et al. [24] estimated at 2.8 % the incidence of intestinal obstructions following appendectomy; with 1.1 % of patients reoperated during the first five years.

Finally, conservative treatment makes sense, especially in preserving resources, both human and material, during this particular conjuncture that the whole world is currently experiencing because of the COVID-19 pandemic [25].

Recent trials and meta-analyses have assessed NOT, which support primary antibiotic treatment of uncomplicated AA [26], [27], [28]. A summary of articles and key findings are presented in Table 1, Table 2.

**Table 1:** Main RCTs comparing antibiotherapy to appendectomy.

Authors/Location	Primary Endpoint	Patient selection	N pts *ATB* vs *Surg*	ATB protocol	Failure rate at 1year*
Eriksson et al., 1995 [41] Sweden	Succes rate	Clin + Lab + us	40 20 vs 20	IV cefotaxime + tinidazole for 2d followed by oral ofloxacin + tinidazole for 8d	40 %
Styrud et al., 2006 [42] Sweden	Succes rate	Clin + Lab	252 128 vs 124	IV cefotaxime + tinidazole for 2d followed by oral ofloxacin + tinidazole 10d	26 %
Hansson et al., 2009 [43] Sweden	Succes rate	Clin + Lab + us	369 119 vs 250	IV cefotaxime + metronidazole for 1d followed by oral ciprofloxacin + metronidazole for 9d	22 %
Vons et al., 2011 [44] France	30-day peritonitis	CT-scan	239 120 vs 119	IV (if nausea) Amoxicillin-clavulanic acid for 2d followed by oral Amoxicillin-clavulanic acid for 6d	25 %
Salminen et al., 2015 [45] Finlande	Succes rate	CT-scan	530 257 vs 273	IV Ertapenem for 3d followed by oral Levofloxacin + metronidazole for 7d	27 %
Flum et al., 2020 [46] USA	30-day health status	CT-scan	1552 776 vs 776	IV ATB for 1d followed by oral ATB for a total of a 10d course, based on the SSI and IDSA guidelines	40 %

N: number, pts: patients, ATB: antibiotics, Surg: surgery, Clin: clinical signs, Lab: laboratory test, us: ultrasonography, d: days, IV: intravenous, SSI: Surgical Infection Society, IDSA: Infectious Disease Society of America. * Failure rate at 1year in the antibiotic group.

**Table 2:** Most recent meta-analyses comparing antibiotic therapy with appendectomy.

Authors	Studies	N pts	Appendectomy within 1 year*	Treatment effectiveness *ATB* vs *Surg*	Complicated appendicitis‡ *ATB* vs *Surg*
Sallinen et al., 2016 [26]	5	1116	22.6 %	–	4.5 vs 6.5 %
Harnoss et al., 2017 [27]	8	2551	26.5 %	72.6 vs 99.4 %	29 vs 17.4 %
Podda et al., 2019 [28]	20	3618	19.2 %	72.6 vs 93.1 %	21 vs 12 %
Zagales et al., 2022 [66]	12	3703	–	73.3 vs 98.4 %	–

N: number, pts: patients, ATB: antibiotics, Surg: surgery, * Rate of appendectomy within 1 year in the antibiotic group, ‡Rate of complicated appendicitis at time of surgery.

### *For which appendicitis?*

Recent data suggest that antibiotic therapy may be considered first-line and potentially unique therapy in some patients with uncomplicated appendicitis. Practically all the clinical trials published so far have assessed the efficacy of NOT in uncomplicated appendicitis; which is defined as appendicitis without perforation, abscess or mass formation [18]. According to recent meta-analyses of randomized controlled trials, NOT can be considered in most patients with uncomplicated appendicitis [26,28]. Peltrini et al. [29] in a meta-analysis published in 2021, estimated the prevalence of appendiceal neoplasm after interval appendectomy for complicated appendicitis at 11 %. Consequently, a strict patient selection, excluding complicated forms, is essential to increase the chances of successful NOT and minimize the risk of missing neoplasia. Independent predictive factors for successful NOT were identified; including age less than 60 years, hyperleukocytosis below 12,000/mm, CRP < 60 g/l, a reduced diameter of the appendix, and no complication signs on CT [30,31]. Under such conditions, Hansson et al. [30] estimated a nearly 90 % success rate of antibiotic treatment.

### *Is there any age restriction?*

Apart from the risk of neoplasm, which tends to increase with age, NOT can be considered in both children and adults [1]. Indeed, advanced age seems to be a risk factor not only for appendicitis recurrence, but also for appendiceal neoplasm [32]. However, this risk remains low, around 1 % and seems to be correlated with complicated forms [29,33,34]. We do not know whether the results of the APPAC trial after five years follow-up should be reassuring; since Salminen et al. [35] found no tumors in the patients from the antibiotic group who underwent surgery; while four tumors were diagnosed in the surgery group. In Contrast, the CODA trial authors found appendiceal neoplasm in seven participants from the appendectomy group and four in the Abx group who underwent appendectomy [36]. Consequently, given the risk of missed tumors is not negligible, it is essential to notify all patients receiving antibiotic treatment of this potential risk.

### *How about pregnant woman?*

Besides the diagnostic challenges, AA in pregnancy is associated with increased morbidity mainly due to perforation. Cases of uncomplicated AA successfully treated medically have been published as Case Reports [37,38]. In a prospective study of 20 pregnant women treated with antibiotics, Joo et al. [39] reported a failure rate of 25 %. Furthermore, several antibiotic therapy protocols have been published. Therefore, and in the lack of high level evidence studies, World Society of Emergency Surgery (WSES) does not recommend the NOT in pregnant women [1].

### *Which imaging for patient selection?*

The uncomplicated status of AA is a prerequisite for any conservative approach. Therefore, the purpose here is not only the diagnosis of AA but also to rule out complicated forms. It should be noted that guidelines do not clearly state how to differentiate complicated from uncomplicated AA [1]. Imaging appears to be an essential tool for making this distinction [40]. First RCTs randomized patients on purely clinical criteria, which probably disadvantaged antibiotic treatment by including complicated cases [41], [42], [43]. In contrast, the authors of the last three RCTs used imaging to better identify complicated AA prior to any randomization [44], [45], [46]. The ultrasound's discriminatory capacity has been evaluated through a prospective study including patients with suspected AA who were scheduled for surgery [47]. The accuracy for detecting complicated appendicitis was significantly higher in children than in adults: sensitivity 41.2% vs. 26.4 % and specificity 94.6% vs. 93.4 %, respectively, *p* = 0.003 [47]. A recent systematic review and meta-analysis by Bom et al. [48] compared all existing studies on imaging methods discriminating complicated from uncomplicated AA. Due to lack of data, summary estimates could only be calculated for CT. The mean sensitivity and specificity were 78 and 91 %, respectively, giving a PPV of 74 % and an NPV of 93 % for detection of complicated AA [48]. The authors concluded, that a staged imaging-based workup using a conditional CT or MRI strategy poorly discriminates complicated from uncomplicated appendicitis in daily practice [48].

### *Does appendicolith change the way?*

The existence of faecalith seems to increase the risk of appendiceal perforation [44,49]. Indeed, Mällinen et al. [50] in a recent study, showed that appendicolith is an independent predictive factor for perforation as well as for NOT failure. By including appendicitis with faecalith in their trial, Vons et al. [44] reported a strong association between the latter and occurrence of complicated appendicitis, increasing the failure rate of NOT. On the other hand, in the APPAC trial, the authors, despite the exclusion of all appendicoliths identified on CT, could not demonstrate the non-inferiority of NOT compared to surgery [45].

More recently, 1552 patients were randomized in the CODA trial [46], including patients with appendicoliths; the authors showed that the risk of NOT failure was three times higher in presence of an appendicolith during the first 48 h HR=2.9 (95 % CI, 1.9–4.4). This risk appears to decrease over time with HR=1.4 from 48 h to 30 days (95 % CI, 0.8–2.4) and HR=1.1 from 31 days to two years (95 % CI, 0.8–1.6). Another highlight of this study involves the appendicular diameter; the authors reported for the first time a correlation between a diameter of 10 mm or more and recurrences. In the light of these findings, NOT is entirely feasible in such patients, as long as they are aware of and accept a relatively high risk of failure.

### *Which antibiotics?*

Without comparative studies, the choice of an antibiotic remains difficult. This choice must necessarily take into account efficacy, cost, availability and route of administration. In addition, to be effective, an antibiotic must be broad-spectrum targeting the germs incriminated in appendicitis. Betalactamines have been widely used. Vons et al. [44] have chosen Amoxicillin-clavulanic acid, motivated by its simplicity of use. The same antibiotic allowed Di Saverio et al. [51] to achieve an overall recurrence rate of about 14 % after 2 years follow-up. Due to its limited spectrum and the emergence of betalactamase-producing strains, Salminen et al. [45] preferred Ertapenem, which requires only one dose per day and provides broad coverage, but exposes to the risk of selecting resistant germs. Most of the randomized studies adopted intravenous antibiotics for 24 to 48 h followed by oral therapy for a total duration of 7 to 10 days. In their trial, Vons et al. [44] opted for an oral route from the onset, with no real influence on the results. Through the APPAC II study, a multicenter, open-label, non-inferiority randomized controlled trial, Haijanen et al. [52] compared oral moxifloxacin for 7 days versus intravenous ertapenem for 2 days followed by oral levofloxacin and metronidazole for 5 days for the treatment of acute uncomplicated appendicitis confirmed by CT scan. Although the 1-year success rate in both groups was greater than 65 %, the authors failed to demonstrate the non-inferiority of success with oral antibiotics compared with intravenous administration followed by oral antibiotics, as the confidence limit exceeded the non-inferiority margin [53].

### *So, what is the performance of the NOT?*

After one year follow-up, Salminen et al. [35,45] reported a 27 % failure rate, with an increasing trend over the years to 39 % after 5 years follow-up. In the CODA trial [46], the largest randomized clinical trial comparing the efficacy of antibiotic therapy versus appendectomy in the treatment of AA; conducted at 25 US medical centers, more than half of the medically treated patients did not require surgery after 4 years follow-up. In this study, Flum et al. [46] showed that 29 % of patients who had antibiotics as a first-line treatment underwent an appendectomy by 90 days, 40 % by one year and 49 % by four years. Also, the authors stated that 4.5 % of appendectomies were motivated by non-clinical reasons, such as travel, and 14 % had no clear reason. Harnoss et al. [27] in a recent meta-analysis, reported a one-year recurrence rate of 27.4 % after NOT; and a significantly lower success rate than surgical treatment (68.4 vs. 89.8 %). More recently, Podda et al. [28] reported a significantly higher success rate after surgical treatment at 1-year follow-up (93.1% vs. 72.6 %; *P* < 0.01). There again, the one-year recurrence rate in the antibiotics group was 19.2 %. Despite its significant recurrence and failure rate, NOT does not seem to increase the risk of appendicular perforation. In the APPAC trial, among patients who received antibiotics and then had secondary surgery within the first year, there were only 10 % of complicated appendicitis and none was major [45]. This rate will drop further to around 2.3 % in 5 years [35]. In their meta-analysis, Podda et al. [28] observed no significant differences between the two groups in terms of complicated appendicitis rate at surgery (Antibiotics: 21.7% vs Surgery: 12.8 %; *p* = 0.07) or surgical complications (Antibiotics: 12.8% vs Surgery: 13.6 %; *p* = 0.66).

In terms of morbidity, Salminen et al. [35] showed a significantly lower complication rate after NOT compared to surgical treatment (6.5% vs. 24.4 %). However, this should be interpreted with caution, since most appendectomies were performed by laparotomy. This approach may have put the surgical group at a disadvantage; knowing the benefits of laparoscopic appendectomy in terms of reduced postoperative pain, small bowel obstruction, wound infections and length of hospital stay [5,54]. Precisely, these facts were not found in the CODA trial in which 96 % of appendectomies were done laparoscopically [46]. The complication rate was higher in the antibiotics group compared to the appendectomy group (8.1% vs. 3.5 %), most significantly in the subgroup of patients with appendicolith [46]. In 2022, Emile et al. [55] published the results of the Umbrella review which included eighteen systematic reviews. The median failure and complication rates of NOT were 25 % (6.9 % - 37.4 %) and 6.9 % (1 - 32.6 %), respectively. In subgroup analysis of meta-analyses that included only adults and RCTs, NOT was associated with higher treatment failure. However, risk of complications was similar between NOT and appendectomy.

In summary, the literature seems to show that even though NOT was associated with a higher failure rate, there was no real impact on the risk of complications since it was similar between NOT and appendectomy. Moreover, NOT has some advantages, including lower rate of postintervention complications and decreased healthcare costs [28].

In terms of financial impact, a recent economic evaluation showed that NOT was associated with a significantly lower cost of care [56,57]. More recently, a cost-utility analysis was conducted in the UK by the National Health Service to compare appendectomy with antibiotic treatment alone [58]. This analysis, based on a non-randomized sample showed that for each quality adjusted life years achieved by using antibiotics alone, an additional GBP 23,278.51 was saved; and thus, suggests that antibiotics are significantly more cost-effective than appendectomy.

The long-term follow-up of the cohorts composing the above-mentioned studies also allowed for the evaluation of the quality of life and patient satisfaction. After a mean follow-up of 7 years, using the EQ-5D-5 L questionnaire, Sippola et al. [59] reported a similar quality of life in both groups. However, satisfaction was higher after surgery, with 89 % of patients reporting to be satisfied or very satisfied compared to 74 % in the antibiotics group; influenced by the low satisfaction score in patients who had to undergo a subsequent appendectomy. This did not prevent 33 % of these patients from stating their wish to opt for a first-line antibiotics treatment again. The results of the CODA trial further supported the fact that antibiotics were non-inferior to appendectomy based on 30-day EQ-5D scores [46].

### *What is the place of NOT in the COVID-19 setting?*

The health care system is by far the most affected by the COVID-19 pandemic. Its impact on surgical practice has been major, with the cancellation of all elective surgery resulting in a disruption of the care chain with a significant decrease in the volume of performed operations [60]. The management of AA during the COVID-19 pandemic experienced a significant increase in the rate of NOT adoption compared to the pre-COVID period. A recent meta-analysis including 2140 patients showed that NOT was applied during the COVID-19 epidemic six times more than before [61]. The mean failure rate of NOT was 16.4 %, more frequent in children and in complicated appendicitis. In addition, the complication rate was significantly lower after NOT compared to appendectomy. The authors concluded that NOT in the context of COVID-19 could be a safe alternative to surgery, with an acceptable failure and complication rate [61].

### *What's beyond the antibiotics?*

Some authors have went even further, Park et al. [62] questioned the need for antibiotics in uncomplicated AA; reporting the notion of spontaneous resolution with only supportive care. In fact, the failure rate in the supportive care group was similar to that in the antibiotics group, as was the perforation rate. The role of antibiotics in AA resolution has been evaluated, recently through the multicenter superiority trial APPAC III, comparing antibiotic therapy with placebo in the treatment of CT scan-confirmed uncomplicated AA [63]. The 10-day treatment success rate was 87 % (95 % c.i. 75–99) and 97 % (92–100) for placebo and antibiotics, respectively. For the primary outcome, this 10 % clinical difference (90 % c.i.−0.9 to 21) was not statistically different (1-sided *P* = 0.142), idem for the secondary endpoints which were similar [64]. This suggests the need for a non-inferiority trial against placebo in the same population, whose results will probably initiate a paradigm shift. It will also have a huge impact on the way we prescribe antibiotics, especially in this time of health crisis, adding to that the emergence of multi-resistant bacterial strains.

### *What does the scientific community think?*

Through *Jerusalem Guidelines,* the WSES considers antibiotic therapy as a safe alternative to surgery and authorizes it as a first-line treatment for CT scan-confirmed uncomplicated AA [1]. The *American College of Surgeons*, based on the results of the CODA trial [46] and previous studies, has recently modified its recommendations for the treatment of AA in the context of the COVID-19 pandemic; qualifying antibiotic therapy "acceptable first-line treatment". This trend is expected to continue even after the end of the pandemic [65].

## Conclusion

First-line NOT seems to be a reasonable approach for the treatment of uncomplicated CT-confirmed AA. When opting for NOT, patients must be informed and be aware of the risk of failure, which is a real Achilles' heel, and may affect almost half of the cases at 5 years. Nevertheless, this approach would allow to successfully treat around three quarters of the patients during the first year. A careful patient selection would undoubtedly improve the success rate. On the other hand, less morbidity, shorter duration of physical disability, and lower overall care costs seem to be the strengths of NOT. In the near future, further studies will be needed to select the best candidates.

## Dissemination of results

Not applicable.

## Authors’ contribution

Authors contributed as follow to the conception or design of the work; the acquisition, analysis, or interpretation of data for the work; and drafting the work or revising it critically for important intellectual content: **HB contributed 100 %**

The author approved the version to be published and agrees to be accountable for all aspects of the work.

## Funding

The author received no funding support for the research, authorship and publication of this manuscript.

## Data availability

Data were derived from published articles.

## Declaration of competing interest

The author declared no conflicts of interest.
